# Improvement of Health-Promoting Functionality of Rye Bread by Fortification with Free and Microencapsulated Powders from *Amelanchier alnifolia* Nutt

**DOI:** 10.3390/antiox9070614

**Published:** 2020-07-13

**Authors:** Sabina Lachowicz, Michał Świeca, Ewa Pejcz

**Affiliations:** 1 Department of Fermentation and Cereals Technology, Wrocław University of Environmental and Life Science, Chełmońskiego 37 Street, 51-630 Wroclaw, Poland; ewa.pejcz@upwr.edu.pl; 2 Department of Biochemistry and Food Chemistry, Agricultural University, Skromna 8, 20-704 Lublin, Poland; michal.swieca@up.lublin.pl

**Keywords:** rye bread, microencapsulation, phenolics, in vitro relative bioaccessibility, lipoxygenase, cyclooxygenase, acetylcholinesterase, biological activity

## Abstract

This study established the appropriate amounts of a functional Saskatoon berry fruit powder in fortified rye bread acceptable to consumers and determined the potential relative bioaccesibility of bioactive compounds exhibiting antioxidant activity, and enzymatic in vitro inhibitory activity against lipoxygenase, cyclooxigenase-1, cyclooxigenase-2, acetylcholinesterase, pancreatic lipase α-glucosidase, and α-amylase, as well as the relative digestibility of nutrients. The content of polyphenolic compounds and antioxidant capability were strongly, positively correlated with the content of the functional additive. The highest phenolics content and antioxidant activity were determined in the products enriched with the powders microencapsulated with maltodextrin (an increase by 91% and 53%, respectively, compared with the control). The highest overall acceptability was shown for the products with 3% addition of the functional additive, regardless of its type. The simulated in vitro digestion released phenols (with the highest bioaccessibility shown for anthocyanins) and enhanced the antioxidant activity of rye bread. In turn, the microencapsulation contributed to the improvement in the relative bioaccesibility of antioxidant compounds. Bread fortification led to an increased inhibitory activity against α-amylase, α-glucosidase, and lipoxygenase. Furthermore, the additive microencapsulated with maltodextrin and inulin improved the capacity to inhibit the activities of pancreatic lipase and cyclooxigenase-2. The results presented allowed concluding that the powders from Saskatoon berry fruits, especially microencapsulated ones, may be a promising functional additive dedicated for the enrichment of rye bread.

## 1. Introduction

Rye bread is an integral element of a man’s diet as a good source of nutrients, including compounds that exhibit a broad spectrum of antioxidant activity. These properties are mainly ascribed to the phenolic compounds of cereal grains [1], which also exhibit anti-carcinogenic and anti-inflammatory activities and are recommended in the prevention of many degenerative diseases [1,2,3]. The consumption of rye bread has increased over the last years also owing to the nutritive value and higher bioaccesibility of its grain components, as well as a lower gluten content [2,3].

The production process of rye bread involves fermentation with sourdough, which positively affects the finished products by acidifying the dough, inhibiting α-amylase activity, developing flavor values, increasing the solubility of pentosans, and also by increasing their nutritive value and antioxidant potential [1,2]. In addition, this technology extends the shelf life of bread by preventing crumb sticking and mold appearance [2].

Worthy of attention is the recent development of the sector of functional food products, which offer increased nutritive and health values that may help prevent many diseases [2,4]. Considering their high intake, food products such as bread can serve as an excellent carrier of functional ingredients that can be introduced at any stage of the technological process. However, when designing fortified/supplemented foods, attention should be paid to their biological value as well as to their sensory profile, including in particular their acceptability by consumers [5]. Investigations conducted so far have demonstrated the fortification of rye bread with grape and tomato pomace to have a positive effect on its nutritive value and its flavor values [4,6]. Considering the properties and composition of Saskatoon berry, a worthwhile alternative to common bread types can be offered by rye bread with the addition of powder from its fruits. Saskatoon berry fruits exhibit a high antioxidant activity that is strongly correlated with the contents of phenolic compounds, vitamins, minerals, free and bound amino acids, and organic acids [7,8,9]. Furthermore, Saskatoon berry fruits exhibit strong antibacterial activity against *Escherichia coli* (VTT E-94564), *Enterococcus hirae* (ATCC 10542), and *Staphylococcus aureus* (VTT E-70045). Their components also exhibit inhibiting activities of α-amylase and α-glucosidase [9,10,11]. The study conducted by Zhao et al. [12] demonstrated that powders from *Amelanchier alnifolia* Nutt. could alleviate hyperlipidemia, hyperglycemia, and blood vessel inflammation induced by high-saccharose and high-fat diet. According to Juricova et al. [7], the Saskatoon berry fruit is additionally responsible for the anti-inflammatory and chemo-protective potency. The sweet taste of the Saskatoon berry fruit can also contribute to the improvement of taste values of sourdough bread by masking its sour taste. On the other hand, establishing the optimal amount of the additive is important as well, because a too high amount of fruit powder can decrease the taste value and cause the appearance of bitterness resulting from a high content of polymerized procyanidins [9].

Another critical issue is the protection of thermolabile bioactive compounds during bread baking at high temperatures. A solution to this problem can be offered by entrapping functional additives in carriers in the microencapsulation process, which allows minimizing losses of health-promoting compounds valuable for a human body. This solution will be worth considering while designing food products with functional characteristics, where not only their production technology, but also their nutritional aspects need to be strongly considered. The effectiveness of microencapsulation has been confirmed in studies into the use of curcumin or Garcinia fruits as functional additives to wheat bread, which demonstrated a higher content of polyphenolic compounds in final products compared with the crude additives [13,14]. The cited authors demonstrated the microencapsulated additive not to affect the complexity of the production process or quality traits of bread, but to contribute to the greater stability of bioactive compounds. According to recent literature, the protection of compounds ensured by the carriers can also contribute to the mitigation of the adverse effects of polyphenolic compounds on sensory properties of the finished products [15] including, for example, the appearance of a bitter and an astringent taste [13]. What is more, the use of microencapsulated food additives has been reported to positively affect the quality traits of bakery products, including bread, by improving their color, dough yield, or even texture [16].

Other important aspects that need to be considered while designing fortified food products include the potential in vitro bioaccesibility of compounds identified in functional additives that are often neglected in the assessment of the real value of such products, as well as the effect their additive has on the nutritive value. As reported by Gawlik-Dziki et al. [17], the quality and quantity of extracted nutrients and their potential health-promoting value determined after simulated in vitro digestion can differ from those obtained for the chemical extracts only. What is more, the bioaccesibility of components of the functional additive will also depend on its interactions (antagonistic or synergistic) with food components or on of its behavior in the digestive system [14]. Therefore, the determination of the potential relative bioaccesibility and biological value of functional food products is essential for the assessment of the effectiveness and safety of the fortification process.

This study examined the effectiveness of rye bread fortification with pure and microencapsulated powders from Saskatoon berry fruits. The study of quality and health-promoting properties was extended with the analysis of potential relative bioaccesibility that indicates the real value of fortified functional food. Furthermore, it aimed to establish the appropriate amounts of a functional additive from Saskatoon berry fruits acceptable by consumers and to determine the antioxidant activity and enzymatic in vitro inhibitory activity against lipoxygenase, cyclooxigenase-1, cyclooxigenase-2, acetylcholinesterase, pancreatic lipase, α-glucosidase, and α-amylase, as well as the relative digestibility of nutrients of fortified rye bread.

## 2. Materials and Methods

### 2.1. Reagents

Acetonitrile, formic acid, methanol, ABTS (2,2′-azinobis(3-ethylbenzothiazoline-6-sulfonic acid), 6-hydroxy-2,5,7,8-tetramethylchroman-2-carboxylic acid (Trolox), 2,4,6-tri(2-pyridyl)-s-triazine (TPTZ), 2,2-Di(4-tert-octylphenyl)-1-picrylhydrazyl (DPPH), methanol, acetic acid, phosphate buffered saline (pH 7.4), LOX Activity Assay kit, AChE Assay kit, α-amylase from porcine pancreas, α-glucoamylase from Rhizopus sp., lipase from porcine pancreas, trini-trobenzenesulfonic acid, NaH_2_PO_4_, and 3,5-dini-trosalicylic acid were purchased from Sigma-Aldrich (Steinheim, Germany). COX Inhibitor Screening Assay Kit was purchased from Cayman (No. 560131; Ann Arbor, MI, USA). (−)-Epicatechin, (+)-catechin, chlorogenic acid, neochlorogenic acid, cryptochlorogenic acid, dicaffeic acid, procyanidin A2, procyanidin B2, *p*-coumaric acid, caffeic acid, 4-caffeoylquinic, kampferol-3-*O*-galactoside, quercetin-3-*O*-rutinoside, quercetin-3-*O*-galactoside, quercetin-3-*O*-glucoside, cyanidin-3-*O*-arabinoside, cyanidin-3-*O*-xyloside, cyanidin-3-*O*-galactoside, and cyanidin-3-*O*-glucoside were purchased from Extrasynthese (Lyon, France). Acetonitrile for ultra-performance liquid chromatography (UPLC; gradient grade) and ascorbic acid were from Merck (Darmstadt, Germany). The carriers (30%) applied to produce powders were maltodextrin DE (20–40) and inulin (Beneo-Orafti, Belgium).

### 2.2. Materials

Fruits—“Smoky” cultivar of Saskatoon berry and rye flour type 720—were used in this work. Saskatoon berry was collected in the year 2019 from BIOGRIM company (Wojciechow, Lublin, Poland), and the rye flour was from Good Mills Poland company (Stradunia, Poland). The freeze drying pure fruit powder (FP) as additives was prepared as described previously by Lachowicz et al. [18]. Meanwhile, the freeze drying encapsulated fruit powder with maltodextrin (FPM) and inulin (FPI) as additives was prepared as described previously by Lachowicz et al. [19]. The resulting Saskatoon berry fruit was ground in Thermomix (Wuppertal, Vorkwek, Germany) at 40 °C for 10 min. For encapsulation fruit, 70% of grounded fruit (*w*/*w*) and 30% of carrier (maltodextrin or inulin) were mixed. Furth, grounded fruit and encapsulated fruit were frozen at −80 °C. Next, the freeze drying method of FP and FPM as well as FPI (≈200 g of each sample) was carried out in a freeze dryer (Christ Alpha 1–4 LSC; Germany) for 24 h at a reduced pressure of 65 Pa. The temperature in the drying chamber was −60 °C, while the heating plate reached 30 °C.

### 2.3. Rye Bread Preparation

The rye doughs were made according to a previously description by Pejcz et al. [3]. Dough samples were made using double-phase type. Yeasts (1%), salt (0.5%), and sourdough (50% of rye flour) were used to make rye flour. The rye dough was fermented by LV2 starter cultures. The next step was placing the dough in a baking tin (at 30 °C for last fermentation). Different rye breads were using different content of fruit powder: 1, 2, 3, 4, 5, and 6%. Breads were baked in a laboratory oven (Brabender, Duisburg, Germany) for 35 min/230 °C. Whole baking experiments were performed twice. Each sample of bread after 24 h was freeze-dried and milled for analysis. The rye bread sample without addition was considered to be the control sample. Symbols of samples: rye bread without additives (BRC) and with fruit powder from 1% (BR1) to 6% (BR6); rye bread with fruit powder encapsulated with maltodextrin from 1% (BRM1) to 6% (BRM6); and rye bread with fruit powder encapsulated with inulin from 1% (BRI1) to 6% (BRI6). The amount of encapsulated fruit powders with maltodextrin (FPM) and encapsulated fruit powders with inulin (FPI) added to bread was calculated per the amount of crude fruit powder (FP).

### 2.4. Sensory Attributes and Colour Parameters

The sensory properties of the obtained rye bread with supplementation were determined using a ten-degree hedonic scale: 1—“I do not like it extremely” to 10—“I like it extremely”. The assessment included the following quality attributes such as aroma, taste, crumb, crust colour, texture, and consistency. It was conducted by a group of 22 panelists (11 men and 11 women in the age group of 20–70). Coded samples were provided to the panelists for the evaluation at 20 °C in a plastic plate.

Colour properties such as L*, a*, and b* of control and enriched rye bread were determined with a Konika Minolta CR-400 colorimeter (Wroclaw Poland). Bread samples were analysed against a white ceramic reference plate (L* = 93.92; a* = 1.03; b* = 0.52), and colour parameters L* (lightness or brightness: black = 0, white = 100), a* (greenness = −a*, redness = +a*), and b* (blueness = −b*, yellowness = +b*) values were recorded. Total change in colour of rye bread samples (ΔE*) was calculated as follows:

$$\Delta E*=\sqrt{{\left(L_{0}^{*}-L^{*}\right)}^{2}+{\left(a_{0}^{*}-a^{*}\right)}^{2}+{\left(b_{0}^{*}-b^{*}\right)}^{2}}$$

### 2.5. Extraction Procedures

#### 2.5.1. Digestion In Vitro

In vitro simulated digestion was made on the basis of the method described by Minekus et al. [20]. For gastrointestinal digestion, the freeze-dried rye bread (250 mg) was mixed with PBS buffer (phosphate-buffered saline) (0.5 mL; pH 7.4) and simulated salivary fluid (1 mL). After that, the sample was shaken (10 min/37 °C). Next, the pH of the mixture was changed to pH 3 using 6 M HCl, and the samples were shaken for 120 min at 37 °C. After that, pH of digests was changed to 7 using 1 M NaOH. The samples underwent intestinal digestion in vitro for 120 min. After that, the activity of enzymes was stopped using methanol (1:1 ratio). The samples were centrifuged (at 19,000× *g*/10 min) and used for the test. 

#### 2.5.2. Chemical Extraction

Freeze-dried rye bread samples (1 g) were mixed with 30% of UPLC-grade methanol (10 mL) with 1% of acetic acid. After that, the extract was sonicated for 20 min (Sonic 6D, Polsonic, Warsaw, Poland) and centrifuged (at 19,000× *g*/10 min). Finally, the extract was filtered by hydrophilic PTFE (politetrafluoroetylen) 0.20 μm membrane (Millex Samplicity Filter, Darmstadt, Germany) and used for testing—chemical extract (CE)

#### 2.5.3. Buffer Extraction

Freeze-dried rye bread (~1 g) was mixed with 20 mL of PBS buffer (pH 7.4), and extracted for 1 h. After the extraction, the extracts were centrifuged (6900× *g*/15 min) and the samples were used for analysis—buffer extracts (BE).

### 2.6. Identyfication and Quantyfication of Polyphenolic Compounds

Determination of polyphenolic compounds of freeze-dried rye bread sample was carried out using an ACQUITY ultra performance liquid chromatography system equipped with a photodiode array detector with a binary solvent manager (Waters Corporation, Milford, MA, USA) series with a mass detector G2 Q/Tof Micro mass spectrometer (Waters, Manchester, U.K.) equipped with an electrospray ionization (ESI) source operating in negative and positive modes [21]. Separations of polyphenolic compounds were carried out using a UPLC BEH C18 column (1.7 μm, 2.1 × 100 mm, Waters Corporation, Milford, MA, USA) at 30 °C. The extracts (10 μL) were injected, and the elution was completed in 15 min with a sequence of linear gradients and isocratic flow rates of 0.45 mL min^−1^. The mobile phase consisted of solvent A (2.0% formic acid, *v*/*v*) and solvent B (100% acetonitrile). The program began with isocratic elution with 99% solvent A (0–1 min), and then a linear gradient was used until 12 min, lowering solvent A to 0%; from 12.5 to 13.5 min, the gradient returned to the initial composition (99% A), and then it was held constant to re-equilibrate the column. The analysis was carried out using full-scan, data-dependent MS scanning from *m*/*z* 100 to 1500. Leucine enkephalin was used as the reference compound at a concentration of 500 pg/μL, at a flow rate of 2 μL/min, and the [M – H]^−^ ion at 554.2615 Da was detected. The [M – H]^−^ ion was detected during 15 min analysis performed within ESI–MS accurate mass experiments, which were permanently introduced via the LockSpray channel using a Hamilton pump. The lock mass correction was ±1.000 for the mass window. The mass spectrometer was operated in negative- and positive-ion mode, set to the base peak intensity (BPI) chromatograms, and scaled to 12,400 counts per second (cps) (100%). The optimized MS conditions were as follows: capillary voltage of 2500 V, cone voltage of 30 V, source temperature of 100 °C, desolvation temperature of 300 °C, and desolvation gas (nitrogen) flow rate of 300 L/h. Collision-induced fragmentation experiments were performed using argon as the collision gas, with voltage ramping cycles from 0.3 to 2 V. Characterization of the single components was carried out via the retention time and the accurate molecular masses. Each compound was optimized to its estimated molecular mass [M – H]^−^/[M + H]^+^ in the negative and positive mode before and after fragmentation. The data obtained from UPLC–MS were subsequently entered into the MassLynx 4.0 ChromaLynx Application Manager software (Waters Corporation, Milford, MA, USA). On the basis of these data, the software is able to scan different samples for the characterized substances. The runs were monitored at the following wavelength: flavonol glycosides at 360 nm. The PDA spectra were measured over the wavelength range of 200–800 nm in steps of 2 nm [21]. The results were as mg per 100 g of dry substances (d.s.).

### 2.7. Health-Promoting Properties

#### 2.7.1. Antiradical Capacity

Rye bread (1 g) was mixed with 80% of methanol and water (10 mL) + 1% hydrochloric acid, and incubated for 20 min under sonication (Sonic 6D, Polsonic, Warsaw, Poland). Next, the slurry was centrifuged at 19,000× *g* for 10 min, and the supernatant was filtered through a hydrophilic PTFE 0.20 μm membrane (Merck, Darmstadt, Germany) and used for analysis.

The ABTS method was performed according to the method described by Re et al. [22]. ABTS^•+^ was generated by oxidation of ABTS with potassiumpersulphate. The ABTS^•+^ solution was diluted to an absorbance of 0.7 ± 0.05 at 734 nm. Then, 0.03 mL of extract was mixed with 2.97 mL of ABTS^•+^ solution and left for 6 min at 25 °C. Next, the absorbance was measured at 734 nm using the UV-2401 PC spectrophotometer (Shimadzu, Kyoto, Japan). The results of antiradical capacity were expressed as Trolox equivalents in µmol per g d.s.

The DPPH method was carried out with the method described by Yen and Chen [23]. Then, 0.50 mL of extract was mixed with ethanol (1.5 mL) and DPPH^•+^ solution (0.5 mL), and left for 10 min at 25 °C. Next, the absorbance was measured at 517 nm using the UV-2401 PC spectrophotometer (Shimadzu, Kyoto, Japan). The results of antiradical activity were expressed as Trolox equivalents in µmol per g d.s.

#### 2.7.2. Reducing Potential

The FRAP test was made on the basis of the method described by Benzie and Strain [24]. First, 0.1 mL of extract was prepared with 0.9 mL of clean H_2_O with 3 mL of ferric complex. Next, after 10 min, the absorbance was checked at 593 nm using the UV-2401 PC spectrophotometer (Shimadzu, Kyoto, Japan). The results of reducing activity were expressed as Trolox equivalents in µmol per g d.s.

#### 2.7.3. Ability to Inhibit the Activity of COX 1 and COX-2

The effect of the bread extract on COX-1 and COX-2 (cyclooxygenase-1 and cyclooxygenase-2) activities was tested using COX Inhibitor Screening Assay Kit (Cayman, No. 560131). One unit of inhibitor activity (IU) was defined as the activity inhibiting 1 unit of enzyme activity. The results were expressed in kIU per g of d.s.

#### 2.7.4. Ability to Inhibit the Activity of Lipoxygenase (LOX)

The LOX inhibitory assay was made on the basis of the method described by Axelroad et al. [25] with modifications. LOX activity was tested by BioTek Microplate Readers in absorbance at 234 nm. The reaction mixture contained 0.245 mL 1/15 mol/L phosphate buffer, 0.002 mL of lipoxygenase solution (167 U/mL), and 0.005 mL of inhibitor solution. After preincubation of the mixture at 30 °C for 10 min, the reaction was initiated by adding 0.008 mL 2.5 mmol/L linoleic acid. One unit of LOX activity was defined as the activity oxidizing 0.12 μmole of linoleic acid per 1 min at reaction conditions. One unit of inhibitor activity (IU) was defined as the activity inhibiting 1 unit of enzyme activity. The results were expressed in kIU per g of d.s.

#### 2.7.5. Ability for Inhibit Acetylcholinesterase Activity (AChE)

The effect of the bread extract on AChE was tested by AChE Assay Kit (Sigma-Aldrich, No. CS0003). One unit of inhibitor activity (IU) was defined as the activity inhibiting 1 unit of enzyme activity. The results were expressed in kIU per g of d.s.

#### 2.7.6. Activity of α-Amylase Inhibitors

α-Amylase inhibitor (αA) activity was made on the basis of the method described by Jakubczyk et al. [26]. α-Amylase from hog pancreas (50 U/mg) was dissolved in the 100 mM phosphate buffer (containing 6 mM NaCl, pH 7.0). To measure the α-amylase inhibitory activity, a mixture of 25 μL of α-amylase solution and 25 μL of sample was firstly incubated at 40 °C for 5 min. Then, 50 μL of 1% (*w*/*v*) soluble starch (dissolved in 100 mM phosphate buffer containing 6 mM NaCl, pH 7) was added. After 10 min, the reaction was stopped by adding 100 μL of 3,5-dinitrosalicylic acid (DNS) and was heated for 10 min. The mixture was then made up to 300 μL with double distilled water and absorbance 540 nm was measured using BioTek Microplate Readers. One αA inhibitory unit (AIU) was as the activity of αA that inhibited one unit of enzyme. αA was as AIU/mg of sample.

#### 2.7.7. Activity of α-Glucoamyalse Inhibitors

α-Glucoamyalse inhibitor (αG) activity was made on the basis of the method described by Jakubczyk et al. [26]. Firstly, 10 μL of α-glucosidase (1 U/mL) and 20 μL 1% saccharose were added to 0.5 mL of 0.1 mol/L phosphor buffer, pH 6.8. The reaction was incubated at 37 °C for 5 min, stopped by adding 100 μL of 3,5-dinitrosalicylic acid (DNS), and heated for 10 min. The mixture was then made up to 300 μL with double distilled water and absorbance 540 nm was measured. For the αGIA measurement, 10 μL of α-glucosidase (1 U/mL) and 50 μL of the sample were added to 0.45 mL of 0.1 mol/L phosphor buffer pH 6.8. After the incubation at 37 °C for 5 min, 20 μL of 1% saccharose was added. The reaction was incubated at 37 °C for 50 min, stopped by adding 100 μL of 3,5-dinitrosalicylic acid (DNS), and heated for 10 min. The absorbance was tested at 540 nm using BioTek Microplate Readers (Bad Friedrichshall, Germany). One αG inhibitory unit (αGAU) was as the activity of αG that inhibited one unit of enzyme. αGA was as AIU/mg of sample.

#### 2.7.8. Activity of Lipase Inhibitors

Lipase inhibitory (LP) activity was made on the basis of the method described by Jakubczyk et al. [26]. First, 2 μL, 100 mg mL^−1^ was added to 5 μL of the sample and 142 μL of 100 mM potassium phosphate buffer, pH 7.5. After preincubation at 30 °C for 3 min, the reaction was initiated by mixing the reaction mixture with 1 μL of a 100 mM pNPA solution in dimethyl sulfoxide (DMSO). The absorbance was tested at 405 nm using BioTek Microplate Readers. Lipase One LP inhibitory unit (AIU) was as the activity of inhibitor that inhibited one unit of enzyme. LP was as IU/mg of sample.

#### 2.7.9. Theoretical Approach

For a clear picture of the relationships between the activity of sample and bioaccessibility of their phenols, as well as pro-healthy properties, the following parameters were described by Gawlik-Dziki et al. [17].

Phenolics bioaccessibility index (ACP), which is an indication of the bioaccessibility of phenolic compounds [15]:

ACP = C_D_/C_R_

where C_D_ = amount of components after simulated gastrointestinal digestion and C_R_ = amount of components after chemical extraction (raw extract).

The biological bioaccessibility index (BAC), which is an indication of the bioaccessibility of antioxidative compounds [15]:

BAC = A_R_/A_D_

where A_D_ = extract after simulated gastrointestinal digestion and A_R_ = raw extract.

### 2.8. Relative Digestibility (RD)

#### 2.8.1. Relative Digestibility Proteins

The RD of proteins was expressed as the differences in the content of free amino groups (FAGs) evaluated for the samples after the in vitro digestion [27]. The amount of FAG was evaluated using the TNBS method [28]. Rye bread (20 μL) was prepared with 0.2 M NaH_2_PO_4_ buffer (0.980 mL; pH 8.0) and 0.1% TNBS (0.5 mL). After 30 min, the absorbance was tested at 340 nm and the amount of FAG was as L-leucine standard (μg per mL).

#### 2.8.2. Relative Digestibility Starch

The RD of starch was as the difference in the content of reducing sugars (RS) evaluated for the samples after the in vitro digestion [27]. The amount of RS was evaluated by DNSA. Bread (0.2 mL) was prepared with H_2_O (0.3 mL) and DNSA reagent (0.5 mL). Next, the substance was incubated at 100 °C/10 min. After that, the absorbance was tested at 540 nm. The amount of RS was as maltose standard (μg per mL).

### 2.9. Statistical Analysis

All experimental results were mean ± SD of two parallel experiments (*N* = 18 for bioactive compounds, enzymatic activity, and relative digestibility; *N* = 20 for colour parameters). Extractions were repeated three times for all analyzed samples. One and multi-way analysis of variance (ANOVA), Duncan’s multiple range, as well as median test were analyzed by Statistica 12.5 (Kraków, Poland).

## 3. Results and Discussion

### 3.1. Sensory Evaluation and Colour Parameters

**Figure 1:**
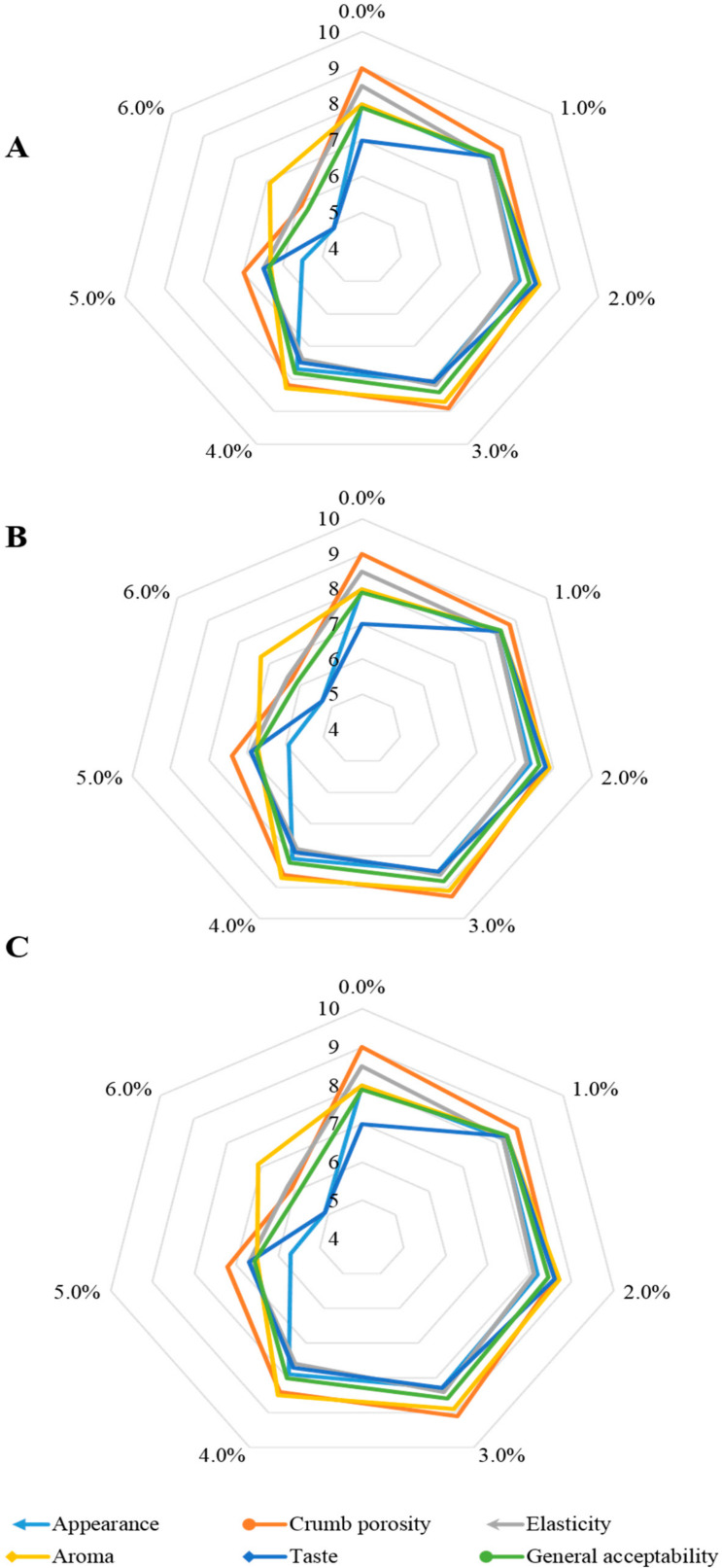
Sensory evaluation of rye bread fortified with fruit powder (**A**), fruit powders covered with maltodextrin (**B**), and fruit powder covered with inulin (**C**).

The sensory assessment of rye breads demonstrated that the use of pure fruit powder (FP), encapsulated fruit powders with maltodextrin (FPM), and encapsulated fruit powders with inulin (FPI) had an insignificant effect on loaf appearance (Figure 1)—the highest scores were given to BRP3, BRM3, and BRI3, as well as to BRM4 and BRI4 breads, whereas the lowest ones were given to the breads with 6% content of the functional additive. In turn, statistically significant differences were noted in terms of aroma and taste, mainly in the breads with 5% and 6% contents of FP, FPM, and FPI compared with the breads with 1% and 4% contents of the functional additive. This happened probably because this content of the additive contributed to the bitter aftertaste and fruity aroma that merged with the acid flavor of rye bread. Deterioration of the sensory traits of breads with berry fruit can also result from a high content of procyanidin polymers that may cause a bitter and astringent aftertaste [13,29]. The highest scores were given by panelists to the breads with 3% and 4% contents of the powders, which were the most effective in making the rye bread taste intensity milder. Considering crumb porosity and elasticity, no statistically significant differences were noted, except for the breads with fruit powder levels of 5% and 6%, in which the values of these parameters slightly decreased. Summing up, the highest score in the overall acceptability assessment was given by the panelists to the bread with 3% content of the functional additive, regardless of functional additive type, as well as to the product with 4% content of the microencapsulated additive. The panelists evaluated this product as more desirable than the control bread. For this reason, the test can be successfully used for the production of sourdough rye breads. In turn, the microencapsulation of the functional additives allowed increasing its content in bread to 4%. This was also confirmed in a study that demonstrated that breads with the addition of a microencapsulated extract from *Garcinia cowa* fruit were scored higher by panelists than the crude fruit extract [13]. Rye breads could also be successfully supplemented with 5% of tomato pomace additive, which improved their taste values [4]. Other investigations have confirmed the feasibility of fortifying rye breads with saffron at levels of 0.08% and 0.12% [30] as well as with grape pomace at the level up to 6% [6], with both additives ensuring product acceptability by consumers.

Saskatoon berry fruit added as a supplement to rye bread (pure powder (FP) and powders encapsulated with maltodextrin (FPM), and inulin (FPI)) significantly diversified the colour of the crumb and crust of rye bread (Table 1). After preparation of rye bread, it was observed that, with the higher amount of FP, FPM, and FPI in rye product production, the L* parameter was lower, which indicates that the enriched rye breads were darker. Thus, BRC was brighter compared with breads with functional additives. In addition, the higher the fruit powder used, the higher the value of the a* parameter and the lower the value of the b* parameter. In turn, the use of encapsulated powder masked the red colour of the fruit powder used. Similar observations of colour were noted by Ezhilarasi et al. [13] for wheat bread supplemented with a fruit powder made from *Garcinia cowa*. The ΔE parameter was calculated for rye bread enriched with additives from pure FP and encapsulated FPM, as well as FPI, and the higher the proportion of additives, the higher the ΔE parameter value. The values of ΔE parameter for crumb bread with 1% and 2% of additives were <5 units, which indicates no difference to distinguish the colours of the two products. Meanwhile, the highest saturation of crust colour was noted in the sample with 6% of additives. Similar observations of change of colour were obtained by Bajerska et al. [30] through the use of saffron to make rye bread. In the case of pure and encapsulate fruit powder, no significant difference in color was noted.

### 3.2. Phenolic Compounds and Their Relative Bioaccesibility

The available literature provides sparse reports on rye bread enrichment with plant material [4,5,6,29]. The effect of rye bread fortification through the addition of pure fruit powder (FP), encapsulated fruit powders with maltodextrin (FPM), and encapsulated fruit powders with inulin (FPI) on the profile and content of polyphenolic compounds is presented in Table 2. Protective effects of carriers on polyphenolic compounds were demonstrated. The content of polyphenolic compounds in powders additionally protected by carriers after freeze-drying was two times higher compared with pure powders (Table S1) [18,19]. Additionally, enriched products in microencapsulated powders after bread baking contained on average a 1.8 times higher content of polyphenolic compounds than breads enriched with pure powder compared with the content of these compounds before baking [18,19]. The total content of polyphenols in BRC reached 127.2 mg/100 g d.s. Already at 1% FP, the mean content of polyphenols was three times higher compared with the control bread, while the 6% addition of crude FP caused a 13-fold increase. In the breads enriched with microencapsulated FPM and FPI, the content of phenols was by ca. 10% and 12% higher than in the breads with crude FP. This could be owing to the protection of these compounds during bread baking. Many authors have confirmed that carriers used in the microencapsulation process protected bioactive compounds from degradation [7,31,32], while the high temperature used during bread baking could contribute to the degradation of phenols in the breads with crude FP. Compared with the breads fortified with crude FP, greater protection of compounds was noted in the group of anthocyanins and in the group of phenolic acids, which reached 11% and 8% as well as 8% and 15% in the breads with FPM and FPI, respectively. These compounds are particularly unstable at high temperatures [18,31,33] and require additional protection that can be ensured by the microencapsulation process. In addition, our observations concerning bread fortification were consistent with earlier reports addressing rye bread enrichment with saffron [30], tomato pomace [4], green tea [5], and grape pomace [6], which demonstrated that the supplementation of rye breads contributed to an increase in the content of polyphenolic compounds.

The use of the functional additive, especially in the form of FPM and FPI, significantly improved the content of polyphenolic compounds in the products examined; however, relative bioaccesibility assessment in the simulated digestive system in vitro was conducted for better identification of the fortification effect in the products with the highest level of the additive (3%) acceptable by consumers (Table 3). Compared with the chemical extracts, the results obtained after simulated in vitro digestion demonstrated that the control sample had a 1.4-fold higher content of phenolic compounds, including a 1.3-fold higher content of flavan-3-ols, as well as a 3.4-fold higher content of phenolic acids, and a 1.2-fold lower content of flavonols. The relative bioaccesibility index computed for BRC showed that phenolic acids were more bioaccessible than the other groups of polyphenolic compounds. In turn, compared with the chemical extracts, the samples of bread enriched with FP obtained after in vitro digestion had a 1.3-fold higher content of polyphenolic compounds, including 5.5-fold and 1.2-fold higher contents of anthocyanins and flavonols, as well as 1.7-fold and 1.2-fold lower contents of phenolic acids and flavan-3-ols, respectively. Analogously, the breads fortified with FPM contained 2-fold higher amount of polyphenols, including 6.1, 1.1, 1.4, and 1.3 times more anthocyanins, flavan-3-ols, phenolic acids, and flavonols, respectively, compared with the chemical extracts. In turn, the breads supplemented with FPI were also characterized by a 2-fold higher content of phenols, including 6.4-fold, 1.2-fold, and 1.3-fold higher contents of anthocyanins, flavan-3-ols, and phenolic acids, respectively, as well as by a 1.2 times lower content of flavonols. The relative bioaccesibility index estimated for the enriched rye bread demonstrated that anthocyanins were highly bioaccessible, and that flavan-3-ols and phenolics were poorly bioaccessible upon the use of FP. The highest relative bioaccesibility index was computed for the breads with FPM addition followed by those with FPI addition, which may indicate that these carriers, and maltodextrin in particular, contributed to greater release of the compounds tested during in vitro digestion. This observation can be explained by better protection of these compounds during bread baking. Besides, it has been described earlier that the digestion process itself can enhance the release of phenolic compounds [34,35], while the low relative bioaccesibility of the compounds could suggest interactions with bread matrix components, which had earlier been reported for the wheat bread enriched with green coffee grains or with broccoli sprouts [17]. The results obtained may also suggest that the use of the powdered functional additive increased the content of phenols, which affected a higher relative bioaccesibility of the rye bread examined, which is indicative of the additive’s effectiveness. In contrast, the microencapsulated functional additive was more effective than the additive without the carrier in increasing the potential relative bioaccesibility of the compounds tested. This was also confirmed by Vitaglione et al. [14], who demonstrated that the fortification of wheat bread with microencapsulated curcumin contributed to greater relative bioaccesibility of bioactive compounds compared with the non-microencapsulated material. According to the authors above, this could be owing to the impaired interactions between the compounds tested and bread matrix. However, the mechanisms of action of the microencapsulated functional additives during simulated in vitro digestion of rye bread require further extensive research. Such attempts will be undertaken in the future.

### 3.3. Pro-Healthy Potency and Their Bioaccesibility

The antioxidant activity of BRC analyzed in ABTS, DPPH, and FRAP tests reached 13.09, 3.04, and 21.00 µmol Trolox/g d.s., respectively (Table 4), and was consistent with the literature data [3]. The addition of Saskatoon berry fruit powder improved the antioxidant potential of the fortified breads compared with BRC. Even the 1% addition of FP, FPM, and FPI to rye bread increased its antiradical activity and reducing properties by 5%, 7.5%, and 6%; 6%, 7%, and 6.5%; as well as by 6%, 8%, and 6.2%, respectively. In turn, the 6% addition of FP, FPM, and FPI increased both the reducing (FRAP) and antiradical potential (ABTS and DPPH) by 39%, 46%, and 46.5%; 39%, 46%, and 47%; as well as by 40%, 47.5%, and 48%, respectively. The increased antioxidant potential of the supplemented rye breads could be owing to sourdough fermentation, because—as reported by Banu et al. [2]—it can increase the extractability of polyphenols from both the additive and rye flour. The baking process of rye bread can also contribute to an increase of its antioxidant potential owing to the appearance of Maillard reaction products [36]. Compared with the use of crude FP, rye bread fortification with microencapsulated FPM and FPI contributed to the greater protection of compounds exhibiting antioxidant potential by 9% and 8% on average in the FRAP assay, by 8% and 7% on average in the ABTS assay, and by 13% and 8% on average in the DPPH assay, respectively. However, there were no statistically significant differences between the microencapsulated additives. Earlier studies have demonstrated rye bread supplementation with grape pomace at the level of 10% to cause a 10-fold increase in its reducing potential [6]. In turn, rye bread fortification with 0.16% of saffron resulted in a 1.6-fold increase in its antiradical activity [30]. A green tea extract added at the level of 1.1% caused a 13-fold increase in the antioxidant activity of rye bread compared with the control sample [5]. As in the study by Mildner-Szkudlarz et al. [6], the higher value of the antioxidant potential was strongly correlated with the amount of polyphenolic compounds, that is, *r*^2^ = 0.801 in the FRAP test and 0.837 in the ABTS test. A similar observation was noted in this work where strong Pearson correlation between polyphenols and antioxidant activity was *r*^2^ = 0.928 for FRAP assay, *r*^2^ = 0.929 for ABTS assay, and *r*^2^ = 0.892 for DPPH assay. In addition, in accordance with the findings reported by Ezhilarasi et al. [13], the microencapsulated functional additives from Garcinia fruits ensured 2-fold greater protection of the antioxidant activity in wheat bread compared with the non-microencapsulated ones. The use of microencapsulated red grape seeds also caused a two times higher antioxidant potential of wheat cookies compared with the product containing a crude additive from grape seeds [37]. The higher antiradical and reducing properties can be owing to the greater protection of bioactive compounds during high-temperature baking [13,37].

However, the antioxidant potential of the bioaccessible fraction of the breads with 3% content of the additive was significantly higher compared with that of the chemical extracts, that is, by 20%, 23%, and 50% in the case of BRP3; by 63%, 27%, and 64% in the case of BRM3; and by 54%, 26%, and 53% in the case of BRI3, while measured with the FRAP, ABTS, and DPPH tests, respectively (Table 5). The analyses demonstrated that in vitro digestion could affect the release of polyphenols from a bread matrix, thereby leading to an increase in their antioxidant activity [33]. Similar results were obtained upon wheat bread enrichment with flaxseed hulls [38] and green coffee extracts [35]. The value of the relative bioaccesibility index computed for the antioxidant activity pointed to high in vitro relative bioaccesibility of these breads. In the FRAP test, the highest value of the relative bioaccesibility index was demonstrated for BRI3 (2.36), whereas the lowest one was for BRC (0.71), while in the ABTS and DPPH test, the highest value of this index was shown for the products containing the functional additive microencapsulated with maltodextrin (3.15 and 2.78). In turn, earlier studies have shown that interactions of bioactive compounds with the matrix of food products can reduce their antioxidant potential [27], which was noted in the case of BRC in our study. In turn, the highest relative bioaccesibility of the microencapsulated functional additives can be owing to the better release of compounds with the antioxidant activity during in vitro digestion or to the protective effect on the formation of complexes with rye bread components. Similar observations were made for the wheat bread enriched with microencapsulated curcumin [14].

Many of the previous studies have demonstrated the in vitro digestion to act as an extractor of compounds with potential relative bioaccesibility and capable of inhibiting enzymes responsible for the induction of inflammatory conditions, including the activity of lipoxygenase (LOX) [17,39]. The effect of in vitro digestion on the selected activities of bread with the optimal content of the functional additive (3%) is presented in Table 5. The highest ability to inhibit LOX activity in the buffered extracts was determined in BRP3 samples, and it was 1.2 times higher compared with BRC. There were no statistically significant differences in the capability for LOX activity inhibition between various forms of the additive (BRC, BRM3, and BRI3). The in vitro digestion caused an increase in the ability to inhibit LOX activity by ca. 29%, with the highest increase noted for BRP3 (over 50%). The relative bioaccesibility index pointed to a high relative bioaccesibility of the supplemented breads, that is, 2.04 (BRP3) 1.25 (BRM3), and 1.37 (BRI3), whereas poor relative bioaccesibility was demonstrated for BRC (0.56). It can be hypothesized that the breads fortified with the fruit powder can suppress the generation of reaction oxygen species (ROS) at the lipoxygenase pathways, thus leading to the inhibition of inflammatory conditions in the body, particularly in the gastrointestinal tract wherein the ROS can lead to the development of carcinogenic lesions [40].

In addition, the breads with the functional additives were determined for their capability to inhibit cyclooxygenases (COX), compared with BRC. The addition of crude FP to bread caused a 2-fold increase in the ability to inhibit COX-1 activity, whereas no such activity was demonstrated in BRM3 and BRI3 products (Table 5). The in vitro digestion caused a 2.5-fold suppression in COX-1 inhibiting activity of BRC, whereas no such activity was observed in the breads fortified with functional additives. In turn, the anti-inflammatory activity analyzed against the inhibition of COX-2 activity in the fortified breads BRP3, BRI3, and BRM3 was 1.3-fold, 2.1-fold, and 2.5-fold higher, respectively, compared with BRC. In the potentially bioaccessible fraction, the best COX-2 inhibiting effect was achieved for BRM3 and BRI3. The potential to inhibit the activity of COX-2 is significant as it is activated in the event of the inflammatory reaction of the body [41] and is responsible for the synthesis of prostaglandin E2, which promotes the development of carcinogenic lesions [42]. The high anti-inflammatory activity against COX-2 can be owing to a higher content of polyphenolic compounds in the products. It was confirmed in the study conducted by Moschon et al. [43], who noted that the microencapsulated products characterized by a higher content of phenols had a higher biological value, including the anti-inflammatory activity.

The use of Saskatoon berry fruits microencapsulated with inulin had only a negligible effect on AChE activity inhibition (an increase by 5%) compared with BRC, whereas no AChE inhibition was noted in the breads with 3% addition of FP and FPM. In turn, the negligibly higher ability to inhibit the activity of this enzyme determined in the products with FPI addition can be owing to the health-promoting properties of inulin [44]. Unfortunately, this activity was not detected in the fractions obtained after in vitro digestion.

Investigations conducted so far have proved that polyphenolic compounds can inhibit the activities of digestive hydrolases, including the activity of pancreatic lipase, being responsible for dietary fat absorption, as well as activities of α-glucosidase (αG) and α-amylase (αA) responsible for the hydrolysis of carbohydrates [10,12]. All additives used for bread fortification were able to inhibit αA activity. The highest inhibition was demonstrated for BRP3 and BRM3, and it was 2.8 and 2.6 times higher compared with BRI3, and 5.3 and 5.6 times higher compared with BRC (Table 6). The control bread exhibited a marginal ability to inhibit αG. This activity was a dozen times higher in BRP3, BRM3, and BRI3. In turn, the ability to inhibit the activity of pancreatic lipase was demonstrated for BRM3 and BRI3, and it was 1.3 and 1.2 times higher compared with BRC, and 1.3 and 1.4 times higher compared with BRP3. It can be concluded that the use of functional additive had a positive effect on the breads’ ability to inhibit activities of αA and αG. In contrast, only the microencapsulated additives inhibited the activity of pancreatic lipase. The high inhibition of digestive enzymes can be owing to the presence of polyphenolic compounds in the additives. This was confirmed by Zhang et al. [45], who noted a strong correlation between the amount of polyphenols and the anti-diabetic activity of lentil. However, an undesirable effect was observed in the rye breads fortified with the microencapsulated powder, which involved the reduction of the potential to inhibit activities of αG and αA compared with FP. It is likely that the carrier can be responsible for masking enzyme inhibitors (polyphenols) and inhibiting their interactions. Considering the phenolic profile of the analyzed breads, it can be speculated that they effectively influence the inhibition of hyperglycemia and obesity, likewise in the study conducted by Zhao et al. [12], who demonstrated that powders from *Amelanchier alnifolia* Nutt. could alleviate hyperlipidemia, hyperglycemia, and blood vessel inflammation induced by high-saccharose and high-fat diet. In addition, the research carried out by Bajerska et al. [30] proved that rye bread enrichment with saffron could lead to the enhanced secretion of insulin and reduced blood levels of glucose and triglycerides. Furthermore, as shown by the literature data [46], the sourdough rye bread itself displays high anti-diabetic and anti-obesity potentials and, according to results of our study, these effects can be enhanced by rye bread fortification. Nevertheless, these metabolic effects need to be further explored in future research.

### 3.4. Relative Digestibility of Starches and Proteins

Functional additives with a high content of polyphenols usually reduce the relative bioaccesibility of nutrients. Considering the above, analyses were conducted to determine the effect of the functional additives on the relative digestibility of starch and proteins (Table 7). In the case of starch digestibility, it was found to decrease upon rye bread enrichment with FP, FPM, and FPI, with the most significant decrease determined in BRP3 (ca. 25%) and BRM3 (ca. 21%). Similar observations were made upon wheat bread enrichment with green coffee powder [35]. The fortification of wheat bread with sorghum flour also led to a ca. 40% decrease in the relative digestibility of starch [47]. Investigations conducted so far have shown that the reduced digestibility of starch and protein can be attributed to polyphenolic compounds present in the plant material, chlorogenic acid in particular [48]. In turn, the fruit powder with the addition of maltodextrin and inulin only slightly decreased protein digestibility (by ca. 16% and 14%, respectively), whereas the use of FP increased it by 27%. Reduced digestibility of protein and starch was also reported upon pasta enrichment with carob flour [27]. In turn, the decreased digestibility of proteins in the in vitro analyses can be owing to the interactions between bioactive compounds of plant origin and components of the bread matrix, leading to the formation of complexes that can be either completely excreted with digested products or inhibit activities of digestive enzymes in the gastrointestinal tract [27]. In addition, the reduction in the relative digestibility of protein can be owing to the fact that rye bread may be a better substrate for digestive enzymes [2].

## 4. Conclusions

The enrichment of rye breads with fruits offers an effective method for the improvement of their biological value. The addition of Saskatoon berry fruit powders to rye bread caused a significant increase in the content of their polyphenols and their antioxidant activity, compared with the control products. The higher the content of the functional additive, the higher the content of antioxidant compounds and their particular groups. Bread supplemented with 3% of the fruit powder, regardless of its form, was acceptable in terms of its sensory attributes and colour. The simulated in vitro digestion showed that anthocyanins of the supplemented rye bread were highly bioaccessible compounds, whereas the least bioaccessible turned out to be flavan-3-ols. Among the functional additives studied, the highest value of the antioxidant potential and the highest relative bioaccesibility of flavonols, flavan-3-ols, and phenolic acids were achieved in BRM3. The addition of fruits caused an insignificant reduction in the relative digestibility of starch and proteins. Bread fortification led to the enhanced capability for the inhibition of α-glucosidase and α-amylase activities, whereas in the case of BRM3 and BRI3 analyses, it additionally showed the ability to inhibit the activity of pancreatic lipase and cyclooxigenase-2 as well as a low inhibitor activity against acetylcholinesterase. On the basis of the above results, it can be concluded that the Saskatoon berry fruit powders, especially these subjected to the microencapsulation process, are valuable and prospective functional additives that increase the attractiveness and nutritive value of rye bread.

## Figures and Tables

**Table 1:** The colour parameter of enriched rye bread. FP, pure fruit powder; FPM, encapsulated fruit powders with maltodextrin; FPI, encapsulated fruit powders with inulin.

	Shares [%]	Rye Bread Enriched with FP				Rye Bread Enriched with FPM				Rye Bread Enriched with FPI			
		L*	a*	b*	ΔE	L*	a*	b*	ΔE	L*	a*	b*	ΔE
Bread crust/dried fruit	BC	52.3 ± 1.0a ^1^	2.8 ± 0.1e	17.0 ± 0.3a	-	-	-	-	-	-	-	-	-
	B1	49.3 ± 1.0b	3.9 ± 0.1d	14.1 ± 0.3b	4.4 ± 0.1f	50.2 ± 1.0a	3.2 ± 0.1e	14.3 ± 0.3a	3.5 ± 0.1f	49.7 ± 1.0a	3.4 ± 0.1e	14.5 ± 0.3a	3.7 ± 0.1e
	B2	48.1 ± 1.0c	4.0 ± 0.1d	12.0 ± 0.2c	6.6 ± 0.1e	49.4 ± 1.0b	3.7 ± 0.1d	13.5 ± 0.3b	4.7 ± 0.1e	49.1 ± 1.0a	3.8 ± 0.1d	13.9 ± 0.3b	4.6 ± 0.1d
	B3	46.2 ± 0.9d	4.9 ± 0.1c	11.2 ± 0.2d	8.7 ± 0.2d	47.4 ± 0.9d	4.5 ± 0.1c	12.6 ± 0.3c	6.8 ± 0.1c	47.1 ± 0.9b	4.6 ± 0.1b	13.0 ± 0.3c	6.8 ± 0.1b
	B4	44.9 ± 0.9e	5.8 ± 0.1b	9.2 ± 0.2e	11.2 ± 0.2c	48.4 ± 1.0c	4.3 ± 0.1c	13.0 ± 0.3b	5.8 ± 0.1d	47.7 ± 1.0b	4.2 ± 0.1c	12.9 ± 0.3c	6.4 ± 0.1c
	B5	42.8 ± 0.9f	6.6 ± 0.1b	8.2 ± 0.2f	13.5 ± 0.3b	45.5 ± 0.9e	4.8 ± 0.1b	11.4 ± 0.2d	9.0 ± 0.2b	47.5 ± 1.0b	4.7 ± 0.1b	12.6 ± 0.3c	6.8 ± 0.1b
	B6	40.5 ± 0.8g	7.3 ± 0.1a	6.2 ± 0.1g	16.6 ± 0.3a	43.1 ± 0.9f	5.2 ± 0.1a	8.6 ± 0.2e	12.7 ± 0.3a	44.9 ± 0.9c	5.2 ± 0.1a	9.5 ± 0.2d	10.8 ± 0.2a
Bread crumb/dried fruit	BCC	40.5 ± 0.8a	7.5 ± 0.2a	9.1 ± 0.2a	-	-	-	-	-	-	-	-	-
	B1C	39.9 ± 0.8b	6.4 ± 0.1b	7.8 ± 0.2b	1.8 ± 0.1c	40.7 ± 0.8a	5.3 ± 0.1b	7.9 ± 0.2b	2.5 ± 0.1e	40.3 ± 0.8a	5.7 ± 0.1b	8.0 ± 0.2b	2.1 ± 0.1e
	B2C	39.8 ± 0.8b	6.3 ± 0.1b	7.8 ± 0.2b	1.9 ± 0.1c	40.8 ± 0.8a	5.8 ± 0.1a	8.7 ± 0.2a	1.8 ± 0.1f	40.6 ± 0.8a	6.0 ± 0.1a	9.0 ± 0.2a	1.6 ± 0.1f
	B3C	38.4 ± 0.8b	5.7 ± 0.1c	5.7 ± 0.1c	4.4 ± 0.1b	39.3 ± 0.8b	5.2 ± 0.1b	6.4 ± 0.1c	3.7 ± 0.1d	39.1 ± 0.8b	5.4 ± 0.1b	6.6 ± 0.1d	3.6 ± 0.1d
	B4C	38.7 ± 0.8b	5.2 ± 0.1c	5.6 ± 0.1c	4.6 ± 0.1b	41.7 ± 0.8a	3.8 ± 0.1c	7.9 ± 0.2b	4.1 ± 0.1c	41.0 ± 0.8a	3.7 ± 0.1c	7.8 ± 0.2c	4.1 ± 0.1c
	B5C	36.6 ± 0.7c	3.4 ± 0.1e	2.8 ± 0.1e	8.4 ± 0.2a	39.0 ± 0.8b	2.5 ± 0.1d	3.9 ± 0.1e	7.4 ± 0.1a	40.6 ± 0.8a	2.4 ± 0.1e	4.3 ± 0.1e	7.0 ± 0.1b
	B6C	35.9 ± 0.7c	4.8 ± 0.1d	3.2 ± 0.1d	7.9 ± 0.2a	38.2 ± 0.8c	3.5 ± 0.1c	4.5 ± 0.1d	6.5 ± 0.1b	39.8 ± 0.8b	3.4 ± 0.1d	4.9 ± 0.1e	5.9 ± 0.1a

^1^ Values are expressed as the mean (*n* = 20) ± standard deviation. Mean values bearing different letters in the same row denote statistical difference (a > b > c … etc.).

**Table 2:** The content of polyphenols in rye bread [mg/100 g d.s.].

	Compounds			Bread Sample					
				BR1	BR2	BR3	BR4	BR5	BR6
Anthocyanins	cyanidin- -3-*O*-galactoside	ND	FP	18.7 ± 0.2m ^1^	38.3 ± 0.3l	88.2 ± 0.8i	135 ± 1g	195 ± 1f	281 ± 2c
			FPM	60.8 ± 0.5k	69.9 ± 0.6j	107 ± 1h	130 ± 1g	230 ± 2d	322 ± 2a
			FPI	65.9 ± 0.6j	75.8 ± 0.7i	108 ± 1h	136 ± 1g	213 ± 1e	298 ± 2b
	cyanidin- -3-*O*-glucoside	ND	FP	2.5 ± 0.1j	6.8 ± 0.1i	8.7 ± 0.1g	18.1 ± 0.2c	30.0 ± 0.3b	41.8 ± 0.4a
			FPM	6.2 ± 0.1i	7.1 ± 0.1h	10.3 ± 0.1f	11.0 ± 0.1e	12.2 ± 0.1e	14.6 ± 0.1d
			FPI	6.4 ± 0.1i	7.4 ± 0.1h	8.8 ± 0.1g	10.9 ± 0.1e	11.7 ± 0.1e	14.0 ± 0.1d
	cyanidin- -3-*O*-arabinoside	ND	FP	ND	2.3 ± 0.1g	3.6 ± 0.1f	5.5 ± 0.1c	7.5 ± 0.1b	11.1 ± 0.1a
			FPM	3.2 ± 0.1f	3.6 ± 0.1f	4.1 ± 0.1e	4.2 ± 0.1e	5.0 ± 0.1d	6.0 ± 0.1c
			FPI	3.4 ± 0.1f	3.9 ± 0.1f	4.2 ± 0.1e	4.4 ± 0.1e	4.7 ± 0.1e	5.6 ± 0.1c
	cyanidin- -3-*O*-xyloside	ND	FP	ND	ND	4.1 ± 0.1e	5.0 ± 0.1c	7.2 ± 0.1b	10.1 ± 0.1a
			FPM	3.3 ± 0.1g	3.8 ± 0.1f	4.1 ± 0.e1	4.6 ± 0.1d	4.4 ± 0.1d	5.2 ± 0.1c
			FPI	3.1 ± 0.1g	3.6 ± 0.1f	3.7 ± 0.1f	3.6 ± 0.1f	4.5 ± 0.1d	5.4 ± 0.1c
	SUM	ND	FP	21.2 ± 0.9n	47.4 ± 1.8m	104 ± 4i	163 ± 6f	240 ± 9e	344 ± 13b
			FPM	73.5 ± 2.8l	84.5 ± 3.3k	125 ± 5h	150 ± 6g	252 ± 11d	348 ± 15a
			FPI	78.8 ± 3.1l	90.6 ± 3.5j	124 ± 5h	155 ± 6g	234 ± 10e	323 ± 14c
Flavan-3-ols	B-type procyjanidin dimer	10.1 ± 0.1o	FP	11.0 ± 0.2n	11.5 ± 0.2m	30.2 ± 0.6g	37.1 ± 0.7f	36.3 ± 0.7f	38.8 ± 0.8e
			FPM	20.6 ± 0.4k	23.7 ± 0.5j	39.4 ± 0.8e	49.6 ± 1.0d	55.5 ± 1.1c	66.6 ± 1.3a
			FPI	19.1 ± 0.4l	22.0 ± 0.4j	27.7 ± 0.6i	29.6 ± 0.6g	49.9 ± 1.0d	59.9 ± 1.2b
	Epigallocatechin x	12.3 ± 0.1k	FP	25.1 ± 0.5j	32.2 ± 0.6g	37.6 ± 0.8e	39.9 ± 0.8d	43.4 ± 0.9c	44.9 ± 0.9b
			FPM	23.7 ± 0.5j	27.2 ± 0.5i	39.5 ± 0.8d	46.6 ± 0.9b	43.8 ± 0.9c	54.5 ± 1.1a
			FPI	30.4 ± 0.6h	36.2 ± 0.7f	44.6 ± 0.9b	44.4 ± 0.9b	45.1 ± 0.9b	55.8 ± 1.1a
	B-type procyjanidin dimer	16.0 ± 0.2o	FP	31.8 ± 0.6g	30.6 ± 0.6g	41.7 ± 0.8d	49.7 ± 1.0c	55.8 ± 1.1b	53.8 ± 1.1a
			FPM	14.9 ± 0.3n	17.1 ± 0.3m	29.5 ± 0.6h	29.7 ± 0.6h	31.4 ± 0.6g	35.7 ± 0.7e
			FPI	21.2 ± 0.4l	23.2 ± 0.5k	26.5 ± 0.5j	28.0 ± 0.6i	29.3 ± 0.6h	33.6 ± 0.7f
	(+)-Catechin	4.0 ± 0.1m	FP	12.0 ± 0.2l	31.4 ± 0.6h	36.1 ± 0.7f	48.5 ± 1.0d	52.9 ± 1.1c	59.3 ± 1.2b
			FPM	17.3 ± 0.3k	19.9 ± 0.4j	37.1 ± 0.7f	42.0 ± 0.8e	48.6 ± 1.0d	58.3 ± 1.2b
			FPI	19.8 ± 0.4j	22.8 ± 0.5i	34.2 ± 0.7g	42.7 ± 0.9e	52.8 ± 1.1c	63.3 ± 1.3a
	(−)-Epicatechin	9.4 ± 0.3k	FP	11.3 ± 0.2j	14.7 ± 0.3h	15.3 ± 0.3g	19.9 ± 0.4d	21.5 ± 0.4c	28.0 ± 0.6a
			FPM	13.9 ± 0.3i	15.9 ± 0.3	18.4 ± 0.4e	18.1 ± 0.4e	24.8 ± 0.5b	27.8 ± 0.6a
			FPI	13.3 ± 0.3i	15.3 ± 0.3	17.7 ± 0.4f	18.3 ± 0.4e	16.4 ± 0.3	19.7 ± 0.4d
	SUM	51.8 ± 1.0n	FP	91.3 ± 9.6m	120 ± 10k	161 ± 10i	195 ± 12f	210 ± 14d	225 ± 12c
			FPM	90.3 ± 4.1m	104 ± 5l	164 ± 9h	186 ± 13g	204 ± 13e	243 ± 16a
			FPI	104 ± 6l	120 ± 8k	151 ± 10j	163 ± 11h	194 ± 15f	232 ± 19b
Phenolic acids	protokatechumic acid	0.9 ± 0.1j	FP	3.1 ± 0.1h	4.9 ± 0.1g	6.2 ± 0.1e	8.3 ± 0.1c	9.5 ± 0.1b	10.5 ± 0.1a
			FPM	0.9 ± 0.1j	1.1 ± 0.1i	4.8 ± 0.0g	0.7 ± 0.0j	1.0 ± 0.1i	1.2 ± 0.1i
			FPI	6.9 ± 0.1e	7.9 ± 0.1c	5.8 ± 0.1f	5.5 ± 0.1f	5.6 ± 0.1f	6.8 ± 0.1d
	vanilic acid	2.5 ± 0.1d	FP	3.5 ± 0.1b	3.3 ± 0.1b	3.6 ± 0.1a	3.8 ± 0.1a	3.3 ± 0.1b	3.6 ± 0.1a
			FPM	2.0 ± 0.1d	2.3 ± 0.1d	2.1 ± 0.0d	2.0 ± 0.0d	2.1 ± 0.1d	2.5 ± 0.1c
			FPI	1.9 ± 0.1d	2.1 ± 0.1d	2.3 ± 0.1d	2.3 ± 0.1d	2.3 ± 0.1d	2.7 ± 0.1c
	caffeic acid	0.9 ± 0.1g	FP	3.1 ± 0.1f	3.5 ± 0.1f	4.2 ± 0.1e	4.6 ± 0.1e	5.2 ± 0.1d	5.7 ± 0.1d
			FPM	3.1 ± 0.1f	3.6 ± 0.1f	7.3 ± 0.1b	7.3 ± 0.1b	6.9 ± 0.1c	8.3 ± 0.1a
			FPI	5.1 ± 0.1d	5.9 ± 0.1d	7.2 ± 0.1b	7.3 ± 0.1b	7.2 ± 0.1b	8.6 ± 0.1a
	3-*O*- -caffequinic acid	0.2 ± 0.1m	FP	60.1 ± 1.0g	72.2 ± 1.0g	86.6 ± 1.1e	103 ± 1d	106 ± 1c	110 ± 1a
			FPM	72.7 ± 0.7l	83.6 ± 0.8k	103 ± 1i	109 ± 1g	116 ± 0e	140 ± 3b
			FPI	89.1 ± 0.8j	102 ± 1i	106 ± 1h	110 ± 0g	114 ± 0f	137 ± 1c
	ferulic acid	5.0 ± 0.1b	FP	5.2 ± 0.1b	5.2 ± 0.1b	5.7 ± 0.1b	6.1 ± 0.1a	5.4 ± 0.1.0b	5.9 ± 0.1a
			FPM	3.1 ± 0.1f	3.6 ± 0.1d	3.6 ± 0.0d	3.5 ± 0.0e	3.7 ± 0.1d	4.4 ± 0.1c
			FPI	3.1 ± 0.1f	3.4 ± 0.1e	3.4 ± 0.1e	3.5 ± 0.1e	3.3 ± 0.1e	3.9 ± 0.1d
	5-*O*- -caffequinic acid	0.4 ± 0.1	FP	43.3 ± 0.4k	49.5 ± 0.4j	76.6 ± 0.7g	102 ± 1e	115 ± 1c	127 ± 1b
			FPM	58.4 ± 0.5i	67.2 ± 0.6h	102 ± 1e	104 ± 1e	115 ± 1c	138 ± 1a
			FPI	75.2 ± 0.7g	86.5 ± 0.8f	104 ± 1e	107 ± 1d	113 ± 1c	136 ± 1a
	3-*p*-coumaroylquinic acid	0.8 ± 0.1h	FP	3.4 ± 0.1g	4.0 ± 0.1g	6.6 ± 0.1g	17.3 ± 0.2e	39.2 ± 0.4b	43.2 ± 0.4a
			FPM	15.4 ± 0.1f	17.7 ± 0.2e	23.0 ± 0.2d	23.2 ± 0.2d	23.6 ± 0.2d	28.3 ± 0.3c
			FPI	17.4 ± 0.2e	20.0 ± 0.2d	21.6 ± 0.2d	23.5 ± 0.2d	25.2 ± 0.2d	30.2 ± 0.3c
	4-*O*- -caffequinic acid	0.2 ± 0.1j	FP	2.2 ± 0.1i	4.9 ± 0.1g	7.3 ± 0.1e	12.1 ± 0.1b	14.1 ± 0.1a	15.4 ± 0.1a
			FPM	3.5 ± 0.1i	4.0 ± 0.1g	5.8 ± 0.1f	7.2 ± 0.1e	8.3 ± 0.1d	10.0 ± 0.1c
			FPI	3.3 ± 0.1i	3.8 ± 0.1h	7.7 ± 0.1e	8.1 ± 0.1d	8.4 ± 0.1d	10.1 ± 0.1c
	SUM	10.9 ± 0.2l	FP	124 ± 2o	148 ± 3n	197 ± 4k	259 ± 5g	298 ± 6c	322 ± 6b
			FPM	159 ± 3m	183 ± 4l	253 ± 5h	257 ± 5g	277 ± 6e	333 ± 7a
			FPI	202 ± 4j	232 ± 5i	259 ± 5g	268 ± 5f	280 ± 6d	336 ± 7a
Flavonols	Kaempferol-3-*O*-galactoside	3.1 ± 0.1j	FP	3.3 ± 0.1j	7.3 ± 0.1h	12.6 ± 0.3f	21.5 ± 0.4c	26.3 ± 0.5b	31.5 ± 0.6a
			FPM	6.5 ± 0.1i	7.4 ± 0.1h	10.9 ± 0.2g	14.0 ± 0.3f	16.3 ± 0.3e	19.2 ± 0.4d
			FPI	7.5 ± 0.1h	8.4 ± 0.2h	10.3 ± 0.2g	13.3 ± 0.3f	13.9 ± 0.3f	16.7 ± 0.3e
	Quercetin-3-*O*-arabinoglucoside	4.2 ± 0.1j	FP	1.8 ± 0.1f	2.3 ± 0.1e	2.6 ± 0.1d	2.9 ± 0.1d	3.6 ± 0.1b	4.0 ± 0.0a
			FPM	2.5 ± 0.1e	2.9 ± 0.1d	3.4 ± 0.1c	3.4 ± 0.1c	3.7 ± 0.1b	4.2 ± 0.1a
			FPI	2.5 ± 0.1e	2.9 ± 0.1d	3.3 ± 0.1c	3.3 ± 0.1c	3.7 ± 0.1b	4.1 ± 0.0a
	Kaempferol-3-*O*-glucoside	32.0 ± 0.3g	FP	34.0 ± 0.7f	36.0 ± 0.7e	37.9 ± 0.8d	42.6 ± 0.9c	48.1 ± 1.1b	51.0 ± 1.1b
			FPM	34.3 ± 0.7f	39.1 ± 0.8d	40.8 ± 0.8c	48.1 ± 1.0b	49.0 ± 1.0b	53.0 ± 1.1a
			FPI	33.5 ± 0.7f	35.3 ± 0.7e	37.1 ± 0.7d	41.8 ± 0.8c	41.9 ± 0.8c	50.3 ± 1.0b
	Quercetin-3-*O*-rutinoside	3.1 ± 0.1h	FP	3.3 ± 0.1g	7.3 ± 0.1f	10.6 ± 0.1d	11.5 ± 0.1c	12.3 ± 0.1b	15.2 ± 0.1a
			FPM	6.0 ± 0.1e	7.1 ± 0.1d	9.7 ± 0.1b	9.4 ± 0.1c	12.2 ± 0.1a	13.6 ± 0.1a
			FPI	6.0 ± 0.1e	6.9 ± 0.1d	9.5 ± 0.1c	11.8 ± 0.1a	12.0 ± 0.1a	13.4 ± 0.1a
	Quercetin-3-*O*-robinobioside	1.1 ± 0.1j	FP	2.5 ± 0.1i	3.5 ± 0.1g	3.7 ± 0.1g	8.1 ± 0.1d	10.0 ± 0.1b	11.3 ± 0.1a
			FPM	3.1 ± 0.1h	3.6 ± 0.1g	6.1 ± 0.1f	6.1 ± 0.1f	8.4 ± 0.1d	9.4 ± 0.1c
			FPI	3.0 ± 0.1h	3.5 ± 0.1g	6.1 ± 0.1f	5.9 ± 0.1f	7.4 ± 0.1e	8.3 ± 0.1d
	Quercetin-3-*O*-galactoside	4.2 ± 0.1h	FP	16.3 ± 0.1i	23.8 ± 0.2h	29.1 ± 0.3g	41.4 ± 0.4e	50.7 ± 0.5b	54.6 ± 0.6a
			FPM	29.7 ± 0.3g	34.1 ± 0.3f	34.3 ± 0.3f	41.6 ± 0.4e	50.8 ± 0.5d	56.8 ± 0.5c
			FPI	31.0 ± 0.3f	35.6 ± 0.3f	34.4 ± 0.3f	51.7 ± 0.5d	59.9 ± 0.5b	67.1 ± 0.6a
	Quercetin-3-*O*-glucoside	ND	FP	4.1 ± 0.1j	4.7 ± 0.1i	6.1 ± 0.1g	7.0 ± 0.1e	7.8 ± 0.1d	8.9 ± 0.1b
			FPM	3.6 ± 0.1k	4.1 ± 0.1j	5.5 ± 0.1h	6.7 ± 0.1f	7.6 ± 0.1d	8.6 ± 0.1b
			FPI	4.1 ± 0.1j	4.7 ± 0.1i	6.6 ± 0.1f	7.9 ± 0.1d	8.1 ± 0.1c	9.1 ± 0.1a
	Quercetin-3-*O*-arabinoside	1.8 ± 0.01g	FP	1.7 ± 0.01g	2.2 ± 0.01f	3.4 ± 0.1d	3.8 ± 0.1c	4.3 ± 0.1b	5.6 ± 0.1a
			FPM	1.9 ± 0.01g	2.1 ± 0.01f	2.2 ± 0.01f	2.6 ± 0.1e	2.7 ± 0.1e	2.9 ± 0.1e
			FPI	1.8 ± 0.01g	1.9 ± 0.01g	2.0 ± 0.01f	2.3 ± 0.1f	2.3 ± 0.1f	2.8 ± 0.1e
	Quercetin-3-*O*-xyloside	ND	FP	2.4 ± 0.1i	3.8 ± 0.1h	4.6 ± 0.1f	5.5 ± 0.1d	6.8 ± 0.1b	7.0 ± 0.a
			FPM	3.9 ± 0.1h	4.5 ± 0.1g	4.8 ± 0.1f	5.2 ± 0.1e	6.1 ± 0.1c	6.9 ± 0.1b
			FPI	4.4 ± 0.1g	5.1 ± 0.1e	5.2 ± 0.1e	5.5 ± 0.1d	5.9 ± 0.1c	6.6 ± 0.1b
	Quercetin-deoxyhexo-hexoside	ND	FP	0.5 ± 0.1c	0.5 ± 0.1c	0.6 ± 0.1b	0.8 ± 0.1a	0.9 ± 0.1a	1.0 ± 0.0a
			FPM	0.3 ± 0.1d	0.3 ± 0.1d	0.4 ± 0.1c	0.5 ± 0.1c	0.8 ± 0.1a	0.9 ± 0.1a
			FPI	0.4 ± 0.1d	0.4 ± 0.1d	0.5 ± 0.1kc	0.7 ± 0.1b	0.7 ± 0.1b	0.8 ± 0.0a
	SUM	43.5 ± 1.3o	FP	69.9 ± 1.4n	88.2 ± 1.8m	106 ± 2	133 ± 3h	155 ± 3e	171 ± 3c
			FPM	91.9 ± 1.8l	105 ± 2k	118 ± 2i	138 ± 3g	158 ± 3d	176 ± 4b
			FPI	94.2 ± 1.9l	105 ± 2k	115 ± 2j	144 ± 3f	156 ± 3e	179 ± 4a
Sum of phenols		106 ± 3o	FP	306 ± 14n	404 ± 18m	568 ± 25j	751 ± 34f	904 ± 43c	1062 ± 56a
			FPM	415 ± 20m	477 ± 23l	661 ±3 2h	731 ± 35g	891 ± 49d	1100 ± 66b
			FPI	479 ± 23l	547 ± 27k	649 ± 32i	730 ± 36g	863 ± 47e	1071 ± 62a

^1^ Values are expressed as the mean (n = 18) ± standard deviation. Mean values bearing different letters in the same row denote statistical difference (a > b > c … etc.). ND—not detected; BC—control rye bread; BR1–BR5—breads with fruit powders (1–6%); FP—additives fruit powders; FPM—additives of encapsulated fruit powders with maltodextrin; FPI—additives of encapsulated fruit powders with inulin.

**Table 3:** Potentially bioaccessible phenolic compounds in the control and enriched rye bread.

	Compounds	Bread Sample				Relative Accessibility Index			
		BRC	BRP3	BRM3	BRI3	BRC	BRP3	BRM3	BRI3
Anthocyanins	ANT1	-	503 ± 2c ^1^	662 ± 2b	695 ± 2a	-	5.7	6.2	6.4
	ANT2	-	34.8 ± 0.1b	45.8 ± 0.2a	48.1 ± 0.2a	-	4.0	4.4	5.4
	ANT3	-	17.3 ± 0.1b	22.7 ± 0.1a	23.8 ± 0.1a	-	4.8	5.6	5.7
	ANT4	-	23.0 ± 0.1b	30.3 ± 0.1a	31.8 ± 0.1a	-	5.7	7.4	8.5
	SUM	-	578 ± 239c	761 ± 315b	799 ± 202a	-	5.5	6.1	6.4
Flavan-3-ols	F301	16.7 ± 0.3d	27.2 ± 0.5c	40.2 ± 0.8a	36.6 ± 0.7b	1.7	0.9	1.0	1.3
	F302	14.2 ± 0.8c	30.5 ± 0.6b	42.7 ± 0.8a	43.3 ± 0.8a	1.1	0.8	1.1	1.0
	F303	13.1 ± 0.6d	5.4 ± 0.1c	30.1 ± 0.6a	23.4 ± 0.7b	0.1	0.1	1.0	0.9
	F304	4.3 ± 0.2d	14.4 ± 0.2c	37.8 ± 0.7b	44.2 ± 0.8a	1.7	0.8	1.0	1.0
	F305	10.2 ± 0.2c	6.1 ± 0.1d	18.7 ± 0.3b	22.8 ± 0.4a	1.1	0.4	1.0	1.3
	SUM	58.5 ± 1.1c	83.7±1.2b	169 ± 3a	170 ± 3a	1.2	0.6	1.0	1.2
Phenolic acid	PA1	2.5 ± 0.1a	1.6 ± 0.1b	2.5 ± 0.1a	2.7 ± 0.1a	2.8	0.3	0.5	0.5
	PA2	5.1 ± 0.1a	0.9 ± 0.1b	1.5 ± 0.1b	1.6 ± 0.1b	2.0	0.3	0.7	0.7
	PA3	12.3 ± 0.1a	5.5 ± 0.1d	8.8 ± 0.1c	9.2 ± 0.1b	15.0	0.8	0.4	0.4
	PA4	5.5 ± 0.1a	2.7 ± 0.1c	4.3 ± 0.1b	4.6 ± 0.1b	5.9	0.7	0.6	0.6
	PA5	2.7 ± 0.1c	121 ± 1b	221 ± 1a	219 ± 1a	17.0	1.0	2.1	2.1
	PA6	5.5 ± 0.1a	2.7 ± 0.1c	4.3 ± 0.1b	4.5 ± 0.1b	1.1	0.5	1.2	1.3
	PA7	0.7 ± 0.1c	63.2 ± 0.3b	99.8 ± 0.4a	95.7 ± 0.4a	1.7	0.8	1.0	0.9
	PA8	1.4 ± 0.1c	6.1 ± 0.1b	9.7 ± 0.1a	10.1 ± 0.1a	7.5	0.8	1.7	1.3
	SUM	35.8 ± 3.7d	204 ± 21c	352 ± 47a	347 ± 39b	3.3	0.9	1.4	1.3
Flavonols	FL1	4.1 ± 0.1b	22.5 ± 0.1a	24.7 ± 0.1a	23.6 ± 0.1a	1.3	1.8	2.3	2.3
	FL2	5.1 ± 0.1b	2.3 ± 0.2a	3.1 ± 0.2a	3.2 ± 0.2a	1.2	0.9	0.9	1.0
	FL3	28.7 ± 0.1c	38.8 ± 0.2b	52.7 ± 0.2a	40.7 ± 0.2b	0.9	1.0	1.3	1.1
	FL4	7.0 ± 0.1b	9.6 ± 0.1a	10.5 ± 0.1a	10.1 ± 0.1a	2.3	0.9	1.1	1.1
	FL5	5.1 ± 0.1b	5.2 ± 0.1a	5.7 ± 0.1a	5.4 ± 0.1a	4.4	1.4	0.9	0.9
	FL6	-	40.3 ± 0.2a	44.3 ± 0.2a	42.3 ± 0.2a	-	1.4	1.3	1.2
	FL7	-	8.1 ± 0.1a	8.9 ± 0.1a	8.5 ± 0.1a	-	1.3	1.6	1.3
	FL8	4.9 ± 0.1d	9.2 ± 0.1a	6.4 ± 0.1b	5.1 ± 0.1c	2.7	2.7	2.9	2.6
	FL9	-	12.6 ± 0.1a	13.8 ± 0.1a	13.2 ± 0.1a	-	2.7	2.9	2.6
	FL10	-	5.4 ± 0.1a	5.9 ± 0.1a	5.6 ± 0.1a	-	2.1	2.5	2.3
	SUM	54.9 ± 9.1d	153 ± 15c	176 ± 18a	157 ± 15b	1.2	1.4	1.5	1.4
Sum of polyphenols		144 ± 25d	1047 ± 217c	1492 ± 270b	1506 ± 292a	1.4	1.6	1.9	2.1

^1^ Values are expressed as the mean (n = 18) ± standard deviation. Mean values bearing different letters in the same row denote statistical difference (a > b > c … etc.). BRC—rye bread control; BRP3—rye bread with 3% of fruit powder; BRM3—rye bread with 3% of fruit powders with maltodextrin; BRI3—rye bread with 3% of fruit powders with inulin; FP—additives fruit powders; FPM—additives of encapsulated fruit powders with maltodextrin; FPI—additives of encapsulated fruit powders with inulin; PC—sum of phenolic compounds; ANT—sum of anthocyanins; FL—sum of flavonols; PA—sum of phenolic acid; F3O—sum of flavan-3-ols (monomers and oligomers); F3O1—B-type procyjanidin dimer; F302—epigallocatechin; F3O3—B-type procyanidin dimer; F3O4—(-)-Epicatechin; F3O5—(+)-Catechin; FL1—kampferol-3-*O*-galactoside; FL2—kampferol-3-*O*-glucoside; FL3—quercetin-3-*O*-arabinoglucoside; FL4– quercetin-3-*O*-rutinoside; FL5—quercetin-3-*O*-robinobioside; FL6—quercetin-3-*O*-galactoside; FL7—quercetin-3-*O*-glucoside; FL8—quercetin-3-*O*-arabinoside; FL9—quercetin-3-*O*-xyloside; FL10—quercetin-3-*O*-deoxy-hexoside; ANT1—cyanidin-3-O-galactoside; ANT2—cyanidin-3-O-glucoside; ANT3—cyanidin-3-O-arabinoside; ANT4—cyanidin-3-O-xyloside; PA1—protocatechuic acid; PA2—vanilic acid; PA3—caffeic acid; PA4—3-*O*-caffequinic acid; PA5—ferulic acid; PA6—5-*O*-*p*-coumaroylquinic acid; PA7, 3-*O*-*p*-coumaroylquinic acid; PA8—4-*O*-*p*-coumaroylquinic acid.

**Table 4:** The antioxidant activity of rye bread fortification with functional additives without and with carriers.

Antioxidant Activity [µmol TE/g ds.]	BRC	Form of Supplement	BR1	BR2	BR3	BR4	BR5	BR6
FRAP	21.0 ± 0.42g ^1^	FP	22.30 ± 0.45f	22.60 ± 0.45e	24.40 ± 0.37d	26.70 ± 0.53c	29.50 ± 0.59b	34.80 ± 0.70a
		FPM	22.70 ± 0.45e	22.90 ± 0.46e	26.50 ± 0.53d	30.20 ± 0.60c	32.30 ± 0.65b	39.00 ± 0.78a
		FPI	22.32 ± 0.33f	23.06 ± 0.40e	25.48 ± 0.45d	28.76 ± 0.58c	32.60 ± 0.65b	39.22 ± 0.78a
ABTS	13.09 ± 0.26g	FP	13.93 ± 0.27f	14.14 ± 0.49c	15.24 ± 0.40e	16.68 ± 0.43d	18.43 ± 0.79b	21.73 ± 0.80a
		FPM	14.19 ± 0.46d	14.32 ± 0.32f	16.58 ± 0.34e	18.84 ± 0.68c	20.27 ± 0.73b	24.39 ± 0.87a
		FPI	13.95 ± 0.35d	14.41 ± 0.35d	15.93 ± 0.33e	17.98 ± 0.63c	20.40 ± 0.73b	24.50 ± 0.87a
DPPH	3.04 ± 0.02k	FP	3.34 ± 0.12j	3.58 ± 0.05i	3.62 ± 0.09i	4.27 ± 0.17g	4.50 ± 0.20f	5.46 ± 0.17d
		FPM	3.70 ± 0.09i	3.66 ± 0.07i	6.94 ± 0.21b	4.51 ± 0.12f	5.14 ± 0.13e	8.51 ± 0.12a
		FPI	3.57 ± 0.15i	3.62 ± 0.17i	4.04 ± 0.08h	4.60 ± 0.19f	5.12 ± 0.09e	5.98 ± 0.10c

^1^ Values are expressed as the mean (n = 18) ± standard deviation. Mean values bearing different letters in the same row denote statistical difference (a > b > c … etc.). BC—control rye bread; BR1–BR6—breads with fruit powders (1–6%); FP—additives fruit powders; FPM—additives of encapsulated fruit powders with maltodextrin; FPI—additives of encapsulated fruit powders with inulin; ABTS—2,2′-azinobis(3-ethylbenzothiazoline-6-sulfonic acid; DPPH—2,2-Di(4-tert-octylphenyl)-1-picrylhydrazyl.

**Table 5:** Ability to inhibit the activity of enzymes related to metabolic syndrome.

In Vitro Potency		Extract	Bread Sample				Relative Accessibility Index			
			BRC	BRP3	BRM3	BRI3	BRC	BRP3	BRM3	BRI3
Antioxidant activity [µmol TE/g d. s.]	ABTS	EBD	13.09 ± 0.26c ^1^	15.24 ± 0.40a	16.58 ± 0.34b	15.93 ± 0.33b	2.20	2.48	3.15	2.72
		PBE	28.78 ± 0.24c	37.44 ± 0.31a	52.24 ± 0.24b	43.37 ± 0.25b				
	DPPH	EBD	3.04 ± 0.02d	3.62 ± 0.09c	6.94 ± 0.21a	4.04 ± 0.08b	1.70	2.00	2.78	2.13
		PBE	5.17 ± 0.39d	7.23 ± 0.21c	19.29 ± 0.72a	8.60 ± 0.28b				
	FRAP	EBD	21.00 ± 0.42c	24.39 ± 0.37d	26.53 ± 0.53a	25.48 ± 0.45b	0.71	1.25	1.84	2.36
		PBE	15.00 ± 0.71d	30.48 ± 0.41c	48.70 ± 0.51b	52.99 ± 0.39a				
Pro-health potency	Inhibition of LOX activity [kIU/g ds]	EBD	1257 ± 30b	1549 ± 2a	121 ± 2c	1116 ± 2d	0.56	2.04	1.25	1.37
		PBF	70.00 ± 0.14c	3168 ± 5d	147 ± 2b	1530 ± 3a				
	Inhibition of COX-1 activity [kIU/g ds]	EBD	4.22 ± 0.84b	8.32 ± 0.66a	-	-	0.37	-	-	-
		PBF	1.56 ± 0.11a	-	-	-				
	Inhibition of COX-2 activity [kIU/g ds]	EBD	7.75 ± 0.55d	10.71 ± 1.14c	19.33 ± 0.39a	15.96 ± 0.32b	0.91	0.36	0.43	0.54
		PBF	7.06 ± 1.41c	3.85 ± 0.77d	8.29 ± 0.66b	8.57 ± 0.71a				
	Inhibition of AChE activity [IU/g ds]	EBD	23.54 ± 0.47b	20.73 ± 0.41d	21.90 ± 0.44c	24.50 ± 0.49a	-	-	-	-
		PBF	-	-	-	-				

^1^ Values are expressed as the mean (n = 18) ± standard deviation. Mean values bearing different letters in the same row denote statistical difference (a > b > c … etc.). EBD—extracts buffer digestion; PBE—potentially bioaccesible fraction; BRC—rye bread control; BRP3—rye bread with 3% of fruit powder; BRM3 —rye bread with 3% addition of fruit powder microcapsulated with maltodextrin; BRI3—rye bread with 3% addition of fruit powder microcapsulated inulin; LOX— lipoxygenase; COX— cyclooxygenases.

**Table 6:** Enzymatic in vitro inhibition activity of enriched rye bread.

Sample	Inhibition of α-Amylase Activity [U/g]	Inhibition of α-Glucosidase Activity [U/g]	Inhibition of Pancreatic Lipase Activity [U/g]
BRC	4.58 ± 0.09d ^1^	0.04 ± 0.01d	1.20 ± 0.02c
BRP3	46.62 ± 0.93a	4.04 ± 0.08a	1.03 ± 0.02d
BRM3	41.58 ± 0.83b	2.32 ± 0.05b	1.27 ± 0.03b
BRI3	16.09 ± 0.32c	2.04 ± 0.04c	1.43 ± 0.03a

^1^ Values are expressed as the mean (n = 18) ± standard deviation. Mean values bearing different letters in the same row denote statistical difference (a > b > c … etc.). BRP3—rye bread with 3% of fruit powder; BRM3—rye bread with 3% of fruit powders with maltodextrin; BRI3—rye bread with 3% of fruit powders with inulin.

**Table 7:** Relative digestibility of starch and proteins.

Bread	Free Reducing Sugars [mg/g]	Released Sugar [mg/g]	Relative Digestibility of Starch	Free Amino Acids and Peptides [mg/g]	Released Amino Acids and Peptides [mg/g]	Relative Digestibility of Proteins
BRC	54.7 ± 1.1d ^1^	473 ± 9a	100 ± 2a	4.4 ± 0.1c	82.3 ± 1.6b	100 ± 2a
BRP3	248 ± 5a	354 ± 7d	75.0 ± 1.5d	6.9 ± 0.1a	105 ± 2a	127 ± 2b
BRM3	158 ± 3b	371 ± 7c	78.4 ± 1.6c	4.2 ± 0.1d	68.5 ± 1.4d	83.3 ± 1.7d
BRI3	100 ± 2c	442 ± 8b	93.4 ± 1.9b	4.8 ± 0.1b	70.8 ± 1.4c	86.0 ± 1.7c

^1^ Values are expressed as the mean (n = 18) ± standard deviation. Mean values bearing different letters in the same row denote statistical difference (a > b > c … etc.). BRC—rye bread control; BRP3—rye bread with 3% of fruit powder; BRM3—rye bread with 3% addition of fruit powder microcapsulated with maltodextrin; BRI3—rye bread with 3% addition of fruit powder microcapsulated inulin.

## Supplementary Materials

The following are available online at https://www.mdpi.com/2076-3921/9/7/614/s1, Table S1: The content of phenolic compounds in pure fruit powder, encapsulated fruit powders with maltodextrin, and encapsulated fruit powders with inulin [mg/100 g d.s.].
