# Supplementary material for: Improvement of Health-Promoting Functionality of Rye Bread by Fortification with Free and Microencapsulated Powders from Amelanchier alnifolia Nutt
Source: Antioxidants (Basel). 2020 Jul 13;9(7):614. doi: 10.3390/antiox9070614 (PMC7402143; doi:10.3390/antiox9070614)
Supplement: Supplementary file 1 [file antioxidants-09-00614-s001.pdf]

antioxidants

Improvement of health-promoting functionality of rye bread by fortification with free and microencapsulated powders from *Amelanchier alnifolia* Nutt.

Sabina Lachowicz [1,*], Michał Świeca [2] and Ewa Pejcz [1]

[1] Department of Fermentation and Cereals Technology, Wrocław University of Environmental and Life Science, Chełmońskiego 37 Street, 51-630 Wroclaw, Poland; sabina.lachowicz@upwr.edu.pl (S.L.); ewa.pejcz@upwr.edu.pl (E.P.);
[2] Department of Biochemistry and Food Chemistry, Agricultural University, Skromna 8, 20-704 Lublin, Poland; michal.swieca@up.edu.pl (M.Ś.)
* Correspondence: sabina.lachowicz@up.wroc.pl (S.L.).

Received: date; Accepted: date; Published: date

Table S1. The content of phenolic compounds in pure fruit powder, encapsulated fruit powders with maltodextrin, and encapsulated fruit powders with inulin [mg/100 g d.s.]

Class of polyphenols	Compounds	FPI	FPM	FP
Flavanols	K-3-gal	42.4±0.8b[2]	50.2±1.0a	19.2±0.4c
	Q-3-ara	3.3±0.1a	3.6±0.1a	1.0±0.01b
	K-3-glu	2.2±0.1a	2.1±0.1a	0.0±0.0b
	Q	0.2±0.1a	0.1±0.1a	0.0±0.0a
	Q-3-rut	43.2±0.9b	46.9±0.9a	27.9±0.6c
	Q-3-rob	0.2±0.01b	0.3±0.01b	1.9±0.01a
	Q-3-gal	201±4b	225±4a	151±3c
	Q-3-glu	9.9±0.2c	11.0±0.2b	19.2±0.4a
	Q-3-ara	3.6±0.1c	5.5±0.1b	6.7±0.1a
	Q-3-xyl	1.8±0.1b	1.9±0.1b	7.7±0.2a
	Q-3-6gal	2.0±0.1b	2.0±0.1b	6.7±0.1a
	Q-3-6glu	6.3±0.1b	7.7±0.2a	2.9±0.1c
	Qdhe	5.2±0.1c	7.7±0.2a	6.7±0.1b
	SUM	322±55b	364±61a	251±40c
Phenolic acids	PrA	6.9±0.1b	9.9±0.2a	1.9±0.1c
	CH	6.6±0.1a	5.7±0.1a	0.0±0.0b
	ThA	22.3±0.4a	23.8±0.5a	24.4±0.5a
	3CQA	319±6b	326±6a	239±4c
	Cag	71.4±1.4a	66.9±1.3b	64.4±1.3c
	5CQA	816±16a	786±15c	791.3±15.8b
	4CQA	200±4a	197±4b	170±3c
	3pCQA	55.8±1.1a	19.7±0.4c	21.2±0.4b
	trans-4CQA	0.0±0.0c	0.0±0.0b	12.5±0.3a
	DCQA	9.0±0.2a	7.6±0.2b	0.0±0.0c
	DCQA	3.4±0.1a	2.8±0.1b	0.0±0.0c

	SUM	1511±246a	1447±240b	1325±236c
Anthocyanins	C-3-hexepi	0.0±0.0b	0.0±0.0b	8.2±0.2a
	C-3-pentepi	0.0±0.0b	0.0±0.0b	3.8±0.1a
	C-3-hexepicat	0.0±0.0b	0.0±0.0b	6.7±0.1a
	C-3-O-gal	1651±33a	1501±30b	1230±24c
	C-3-O-glu	443±8a	423±8b	272±5c
	C-3-O-ara	106±2a	64.1±1.3c	70.2±1.4b
	C-3-O-xyl	159±3a	120±2b	109±2c
	C	2.7±0.1a	2.0±0.1b	0.0±0.0c
	SUM	2363±568a	2111±520b	1701±421c
Flavan-3-ols	PB4	228±4a	204±4b	0.0±0.0c
	PB4	7.4±0.1a	4.6±0.1b	0.0±0.0c
	PA2	79.5±1.6b	67.8±1.4c	100±2a
	PB2	47.6±1c	51.1±1.0b	92.3±1.8a
	Cat	30.4±0.6a	23.8±0.5b	23.1±0.5b
	PB3	40.8±0.8b	55.9±1.1a	55.8±1.1a
	Epi	4.2±0.1c	5.4±0.1b	10.6±0.2a
	PB4	33.0±0.7c	56.4±1.1a	43.3±0.9b
	PB4	4.8±0.1a	4.7±0.1a	2.1±0.1b
	PB3	15.8±0.3	15.9±0.3	25.8±0.5
	PB3	6.3±0.1a	5.7±0.1b	4.8±0.1c
	PB4	29.3±0.6a	29.7±0.6a	6.7±0.1b
	PB4	47.2±0.9a	44.2±0.9b	0.0±0.0c
	SUM	574±59a	569±53b	364±35c
Total phenolic compounds		4772±933a	4492±809b	3642±714c
Antioxidant activity	FRAP	33.1±0.7b	38.0±0.8a	18.0±0.4c
[µmol TE/g d.s.]	ABTS	50.0±1.0a	49.5±1.0a	30.1±0.6b

[1] Values are expressed as the mean (n = 3) ± standard deviation. Mean values bearing different letters in the same row denote statistical difference (a>b>c...etc).

Epi, (-)-epicatechin; Cat, (+)-catechin; PB4, B-type procyanidin tetramer; PB3, B-type procyanidin trimer; PB2, B-type procyanidin dimer; PA2, A-type procyanidin dimer; C-3-gal, cyanidin-3-*O*-galactoside; C-3-glu, cyanidin-3-*O*-glucoside; C-3-ara, cyanidin-3-*O*-arabinoside; C-3-xyl, cyanidin-3-*O*-xyloside; C, cyaniding; PrA, protocatechuic acid; Cag, caffeic acid glucoside; Chx, caffeoylhexose; ThA, trihydroxycinnamoylquinic acid isomers; 3CQA, 3-*O*-caffequinic acid); 5CQA, 5-*O*-caffequinic acid); 4CQA, 4-*O*-caffequinic acid); 3pCQA, 3-*O*-*p*-coumaroylquinic acid; DCQA, di-caffeoylquinic acid; DCQA, di-caffeoylquinic acid; Q, quercetin; Q-3-rob, quercetin-3-*O*-robinobioside; Q-3-rut; quercetin-3-*O*-rutinoside; Q-3-ara; quercetin-3-*O*-arabinoside; Q-3-xyl, quercetin-3-*O*-xyloside; Q-3-glu, quercetin-3-*O*-glucoside; Q-3-gal, quercetin-3-*O*-galactoside; Q-3-arab; quercetin-3-*O*-arabinobioside; Q-3-6glu, quercetin-3-*O*-(6''-acetyl)glucoside; Q-3-6gal, quercetin-3-*O*-(6''-acetyl)galactoside; Qdhe, quercetin-deoxyhexo-heksoside; K-3-gal, kaempferol-3-*O*-galactoside; K-3-glu, kaempferol-3-*O*-glucoside; C-3-hexepi, C-3-hexoside-(epi)catechin; C-3-pentepi, C-3-pentoside-(epi)catechin; C-3-hexepicat, C-3-hexoside-(epi)cat-(epi)cat.

© 2020 by the authors. Submitted for possible open access publication under the terms and conditions of the Creative Commons Attribution (CC BY) license (http://creativecommons.org/licenses/by/4.0/).
